# *Sost* Deficiency does not Alter Bone’s Lacunar or Vascular Porosity in Mice

**DOI:** 10.3389/fmats.2017.00027

**Published:** 2017-09-13

**Authors:** Henry Mosey, Juan A. Núñez, Alice Goring, Claire E. Clarkin, Katherine A. Staines, Peter D. Lee, Andrew A. Pitsillides, Behzad Javaheri

**Affiliations:** 1 Skeletal Biology Group, Comparative Biomedical Sciences, The Royal Veterinary College, London, United Kingdom; 2 Faculty of Natural and Environmental Sciences, Biological Sciences, University of Southampton, Southampton, United Kingdom; 3 School of Applied Sciences, Edinburgh Napier University, Edinburgh, United Kingdom; 4 Manchester X-Ray Imaging Facility, University of Manchester, Manchester, United Kingdom

**Keywords:** *Sost*, osteocyte, vascular porosity, microCT, lacunar porosity

## Abstract

SCLEROSTIN (*Sost*) is expressed predominantly in osteocytes acting as a negative regulator of bone formation. In humans, mutations in the *SOST* gene lead to skeletal overgrowth and increased bone mineral density, suggesting that SCLEROSTIN is a key regulator of bone mass. The function of SCLEROSTIN as an inhibitor of bone formation is further supported by *Sost* knockout (KO) mice which display a high bone mass with elevated bone formation. Previous studies have indicated that *Sost* exerts its effect on bone formation through Wnt-mediated regulation of osteoblast differentiation, proliferation, and activity. Recent *in vitro* studies have also suggested that SCLEROSTIN regulates angiogenesis and osteoblast-to-osteocyte transition. Despite this wealth of knowledge of the mechanisms responsible for SCLEROSTIN action, no previous studies have examined whether SCLEROSTIN regulates osteocyte and vascular configuration *in cortices of mouse tibia*. Herein, we image tibiae from *Sost* KO mice and their wild-type (WT) counterparts with high-resolution CT to examine whether lack of SCLEROSTIN influences the morphometric properties of lacunae and vascular canal porosity relating to osteocytes and vessels within cortical bone. Male Sost KO and WT mice (*n* = 6/group) were sacrificed at 12 weeks of age. Fixed tibiae were analyzed using microCT to examine cortical bone mass and architecture. Then, samples were imaged by using benchtop and synchrotron nano-computed tomography at the tibiofibular junction. Our data, consistent with previous studies show that, *Sost* deficiency leads to significant enhancement of bone mass by cortical thickening and bigger cross-sectional area and we find that this occurs without modifications of tibial ellipticity, a measure of bone shape. In addition, our data show that there are no significant differences in any lacunar or vascular morphometric or geometric parameters between *Sost* KO mouse tibia and WT counterparts. We, therefore, conclude that the significant increases in bone mass induced by *Sost* deficiency are not accompanied by any significant modification in the density, organization, or shape of osteocyte lacunae or vascular content within the cortical bone. These data may imply that SCLEROSTIN does not modify the frequency of osteocytogenic recruitment of osteoblasts to initiate terminal osteocytic differentiation in mice.

## Introduction

Bone is a metabolically active tissue constantly adapting its structure to external mechanical stimuli, leading to changes in mass (Raab et al., 1991; Bennell et al., 2002), shape (Rubin, 1984), strength (Järvinen et al., 2003; Leppänen et al., 2008), and length (Howell, 1917; Steinberg and Trueta, 1981). The exact mechanisms for transformation of these mechanical signals into biological responses are not fully elucidated. Osteocytes, the most abundant cells within skeleton, are derived by a process termed osteocytogenesis from bone-forming osteoblasts. They reside within irregularly shaped ellipsoidal lacunar spaces (Franz-Odendaal et al., 2006) and are reported to act as strain sensors and transducers (Bonewald and Johnson, 2008; Javaheri et al., 2014). There have been a number of hypotheses put forward as to how osteocytes achieve their mechanosensory role. One, the fluid flow hypothesis, states that mechanical loading perturbs bone fluid through the lacunar-canaliculi network producing shear forces on the osteocyte cell processes (Burger and Klein-Nulend, 1999; Han et al., 2004). An alternative proposes that osteocytes are vital for efficient bone remodeling and repair of bone microdamage (Verborgt et al., 2000; Ma et al., 2008). Besides these roles, osteocytes are also reported to regulate matrix mineral homeostasis (Qing and Bonewald, 2009) through osteocytic osteolysis (Bélanger, 1969; Kerschnitzki et al., 2013).

The cellular mechanisms that regulate mechanotransduction are not fully understood, but several key pathways have been identified in osteocytes. One such pathway involves control of the canonical Wnt signaling pathway by its negative regulator (Balemans et al., 2005; Weivoda et al., 2017) SCLEROSTIN; a protein encoded by the *Sost* gene expressed predominantly by deeply embedded mature osteocytes (Balemans et al., 2001).

SCLEROSTIN and the canonical Wnt signaling pathway regulate bone mass through several mechanisms, including stem cell renewal (Reya and Clevers, 2005), stimulation of pre-osteoblast differentiation and proliferation (Kato et al., 2002), enhancement of osteoblast activity (Kato et al., 2002; Bodine et al., 2004), inhibition of osteoblast and osteocyte apoptosis (Babij et al., 2003), regulation of osteoclastogenesis (Glass et al., 2005; Holmen et al., 2005), and modulation of adaptive response to mechanical strain (Armstrong et al., 2007; Javaheri et al., 2014). In addition, previous *in vitro* studies have reported that SCLEROSTIN promotes osteocytogenesis, or osteoblast-to-osteocyte differentiation (Atkins et al., 2011), and angiogenesis in human endothelial cells *in vitro* (Oranger et al., 2017). Moreover, *Sost* deficiency leads to higher matrix mineralization and enhanced bone mass; mineralization has been reported to influence terminal osteoblast differentiation into osteocytes (Cameron et al., 1967; Prideaux et al., 2012) and angiogenesis (Choi et al., 2000; Van Wesenbeeck et al., 2002). It remains unclear whether the role of SCLEROSTIN as a negative regulator of bone mass extends, however, to controlling osteocyte lacunar organization and vascular content in bone.

Visualization of osteocytes and vascular canals is difficult in bone’s highly mineralized matrix. Thus, spatial characteristics, including density, volume, and shape of osteocyte lacunae and vascular porosity have, therefore, often been used as a proxy (Qiu et al., 2003; Carter et al., 2013; Javaheri et al., 2015). This is predominantly due to the fact that 3D quantification of osteocyte and vessel density and morphology is problematic using traditional imaging techniques including confocal microscopy. More recently, nano-computed tomography (nanoCT) has been employed to attain sub-micron resolution of lacuna and vascular porosity using benchtop CT scanners that offer sub-micron scanning. We (Javaheri et al., 2015) and others (Robling and Turner, 2002; Cooper et al., 2004; Mullender et al., 2005; Skedros et al., 2005; Matsumoto et al., 2006; Vatsa et al., 2008; Schneider et al., 2009, 2010; Palacio-Mancheno et al., 2014) have previously reported that nanoCT is a useful tool to quantify osteocyte and vascular porosity in 3D. However, the gold standard for such imaging remains synchrotron-based nanoCT which provides a higher X-ray flux and lower beam hardening effect due to monochromatic beam, which in principle can improve the image quality (Pacureanu et al., 2012; Carter et al., 2013; Dong et al., 2013; Kerschnitzki et al., 2013; Mader et al., 2013; Webster et al., 2013).

To our knowledge, no previous study has investigated SCLEROSTIN’s role in the 3D organization of the osteocyte and vascular networks in bone. We hypothesize that *Sost* deficiency results in altered osteoblast-to-osteocyte transition and vascular network formation as reflected by changes in lacunar properties including numbers, volume, diameter, and shape as well as vascular parameters, respectively. To address this, we employ desktop as well as synchrotron-based nanoCT analyses of lacunar and vascular contents in a *Sost*-deficient mouse model.

## Materials and Methods

### Animal Model

Frozen sperm from a male *Sost* knockout (KO) mouse was purchased from the Knockout Mouse Project Repository at the University of California Davis, CA, USA. Imported sperm used to fertilize ova from C57BL/6 wild-type (WT) mice. To obtain offspring, fertilized egg was implanted into pseudopregnant female C57BL/6 mice. Offspring were bred through several generations to obtain *Sost* homozygous mice on a C57BL/6 background.

Mice were provided with standard mouse chow and water *ad libitum* throughout the study and housed up to four per cage in polypropylene cages with wood chip and paper bedding. Weaners up to 8 weeks of age were fed a standard rodent breeding diet and thereafter a standard rodent maintenance diet (Special Diet Services, South Witham, UK). All procedures performed were reviewed and approved by the ethics committee of the Royal Veterinary College (London, UK) and complied with the UK Animals (Scientific Procedures) Act 1986.

### Imaging

#### High-Resolution Micro and NanoCT

**MicroCT**

Two groups of mice, male *Sost* KO and WT mice (*n* = 6/group), at 12 weeks of age were sacrificed by cervical dislocation. Right tibia from each mouse was dissected, the flesh around the bone removed and fixed in 70% EtOH. Prior to scanning, tibiae were removed from 70% EtOH and dried superficially on paper tissue, before being wrapped in plastic “cling-film,” to prevent drying during scanning, and scanned *ex vivo* using the Skyscan 1176 (Skyscan, Kontich, Belgium), with X-ray tube operated at 50 kV and 600 μA, 2,000 ms exposure time, a rotation step of 0.800°, a 1-mm aluminum filter and a voxel size of 9 μm within a field of view of 11.5 mm (width) and 7.8 mm (height). The slices were then reconstructed using NRecon 1.6.9.4 (Skyscan, Kontich, Belgium). Whole bone analysis was performed on datasets derived from CT scans using BoneJ (Doube et al., 2010) (version 1.4.0) a plugin for ImageJ (Schneider et al., 2012). Following alignment and removal of fibula from the dataset a global bone threshold of 75 Gy level was used to segment bone from non-bone in the images. To determine whether *Sost* deficiency alters cortical bone architecture, we undertook gross bone morphology analysis. The cross-sectional area (CSA), second moment of area around minor (I_min_) and major axes (I_max_) and mean thickness were calculated within BoneJ (“Slice Geometry”). The most proximal and the most distal 10% portions of tibial length included trabecular bone and thus were not included in the analysis.

**Nano-Computed Tomography**

The same tibiofibular junction of the *Sost* KO and WT mice tibiae scanned previously by micro-CT (2.2.1.1), were re-scanned using a Skyscan 1172 (Skyscan, Kontich, Belgium) X-ray microtomography as described previously (Javaheri et al., 2015). This scanner offers isotropic detail detectability down to 0.5 μm. The samples were placed in Orthodontic Wax (Kerr, CA, USA) at 200 μA, 50 kV and, 9,800 ms exposure time with a 0.25-mm aluminum filter (99.999% purity, Goodfellow, Huntington, UK), 360° at a rotation step of 0.25° and a voxel size of 0.6 μm. Scans were centered at the tibiofibular junction within a field of view of 2.3 mm (width) and 1.6 mm (height). Two-frame averaging was used to improve the signal-to-noise ratio. The scan time for each sample was approximately 7 h. Prior to reconstruction, thermal shift in projection images was corrected in NRecon 1.6.9.4 (Skyscan, Kontich, Belgium). 300 slices (0.6 μm per each slide, total 180 μm) were then reconstructed in NRecon using a ring correction factor of 15, smoothing of 1 and 35% beam hardening correction. Three 100 consecutive images from the tibiofibular junction were selected from each specimen. The images were loaded in CTAn software (Skyscan, Kontich, Belgium). The major differences between nanoCT and microCT (2.2.1.1) are the voxel size, exposure time, and angular rotation step.

Initially, foreground was segmented from background and a series of noise removal “despeckling” steps performed. Pores smaller than 13 μm^3^ and larger than 1,500 μm^3^ were assumed to be noise and vascular canals, respectively, and the rest were considered to be lacunae. These limits were based on a previous study using confocal microscopy indicating a size between 28 and 1,713 μm^3^ for osteocytes (McCreadie et al., 2004). Previous studies used and reported these volume limits to examine lacunar and canal porosity (Tommasini et al., 2012; Carter et al., 2013; Carriero et al., 2014; Javaheri et al., 2015). Morphometric indices for lacunar and vascular canals were calculated by measuring the 3D parameters of each discreet object within the volume of interest after segmentation. These indices for lacuna included average lacunar number (N.Lc), average lacunar volume (Lc.Avg.V), total volume (Lc.Tot.V), diameter (Lc.D; calculated with the sphere-fitting method), number of lacunar pores per unit bone volume (N.Lc/Ct.BV), and number of lacunar pores per total volume (N.Lc/Tot.V). For vascular canals, number (Ca.N), total volume (Ca.Tot.V), diameter (Ca.D; calculated with the sphere-fitting method), number of vascular canals per unit bone volume (N.Ca/Ct.BV), and number of vascular canals per total volume (N.Ca/Tot.V) were measured. Shape analysis of the lacunae was conducted utilizing “Analyze Particles” function in BoneJ. Shape parameters were then computed for each ellipsoid based upon the resulting three radii. The best-fit ellipsoid provided lacuna major radius (Lc.λ1), lacuna intermediate radius (Lc.λ2), and lacuna minor radius (Lc.λ3), which correspond to the lacuna’s principal axes (i.e., the eigenvalues of the inertial matrix). These values allowed calculation of the degree of lacunar elongation [Lc.El = 1 − (Lc.λ2/Lc.λ1)] and degree of lacunar flatness [Lc.Fl ≥ 1 − (Lc.λ3/Lc.λ2)] (Javaheri et al., 2015). The composition of the structure was then plotted using a Flinn diagram (Flinn, 1962) showing major: intermediate axis ratio on the *y*-axis and the intermediate: minor axis ratio on the *x*-axis.

#### Synchrotron NanoCT

Tibiae from separate groups of male *Sost* KO and WT mice (*n* = 6/group) were fixed in 70% EtOH and were embedded in wax to prevent sample movement. Tibiofibular junctions were scanned using SR CT at the TOMCAT beamline of the Swiss Light Source at a voxel size of 0.65 μm. This scanner offers isotropic detail detectability down to 0.37 μm. For each scan projection, images were acquired over a range of 360° at a rotation step of 0.12°, a photon energy of 18.5 keV, 18 ms exposure, corrected for ring artifacts due to potential scintillator defects and reconstructed based on gridrec algorithm using graphical user interface written in Python/Jython (Marone and Stampanoni, 2012) and developed as a plugin for Fiji (Schindelin et al., 2012). Scans were centered at the tibiofibular junction (maximum outer dimension of 1.5 mm) within a field of view of 3.3 mm (width) and 1.4 mm (height). Tomographic datasets were typically acquired in 10–15 min. SR CT datasets consisted of a stack of 1,000 reconstructed CT slices, 300 of which were used for morphometric analysis as described previously (see Nano-Computed Tomography).

### Statistical Analysis

Normality and homogeneity of variance were used for all comparisons between *Sost* KO and WT mice. Violations of normality and homogeneity were not observed.

For gross cortical bone morphology analysis, graphs were plotted using the programming language “R,” version 3.1.3 (R Foundation for Statistical Computing, Vienna, Austria; http://www.r-project.org). For this purpose, the functions lattice and grid were used. Two-sample *t*-test was used for comparisons between *Sost* KO and WT mice. Data are presented as mean ± SEM and were considered statistically significant when *p* < 0.05.

Graphs relating to lacunar and vascular porosity data, obtained from either benchtop nanoCT or synchrotron were generated using GraphPad Prism 6 (GraphPad Software, Inc., San Diego, CA, USA). Two-sample *t*-test was used for comparisons between *Sost* KO and WT mice. Data are presented as mean ± SEM and were considered statistically significant when *p* < 0.05.

## Results

### *Sost* Deficiency Produce gross changes in cortical Bone

**Figure 1:**
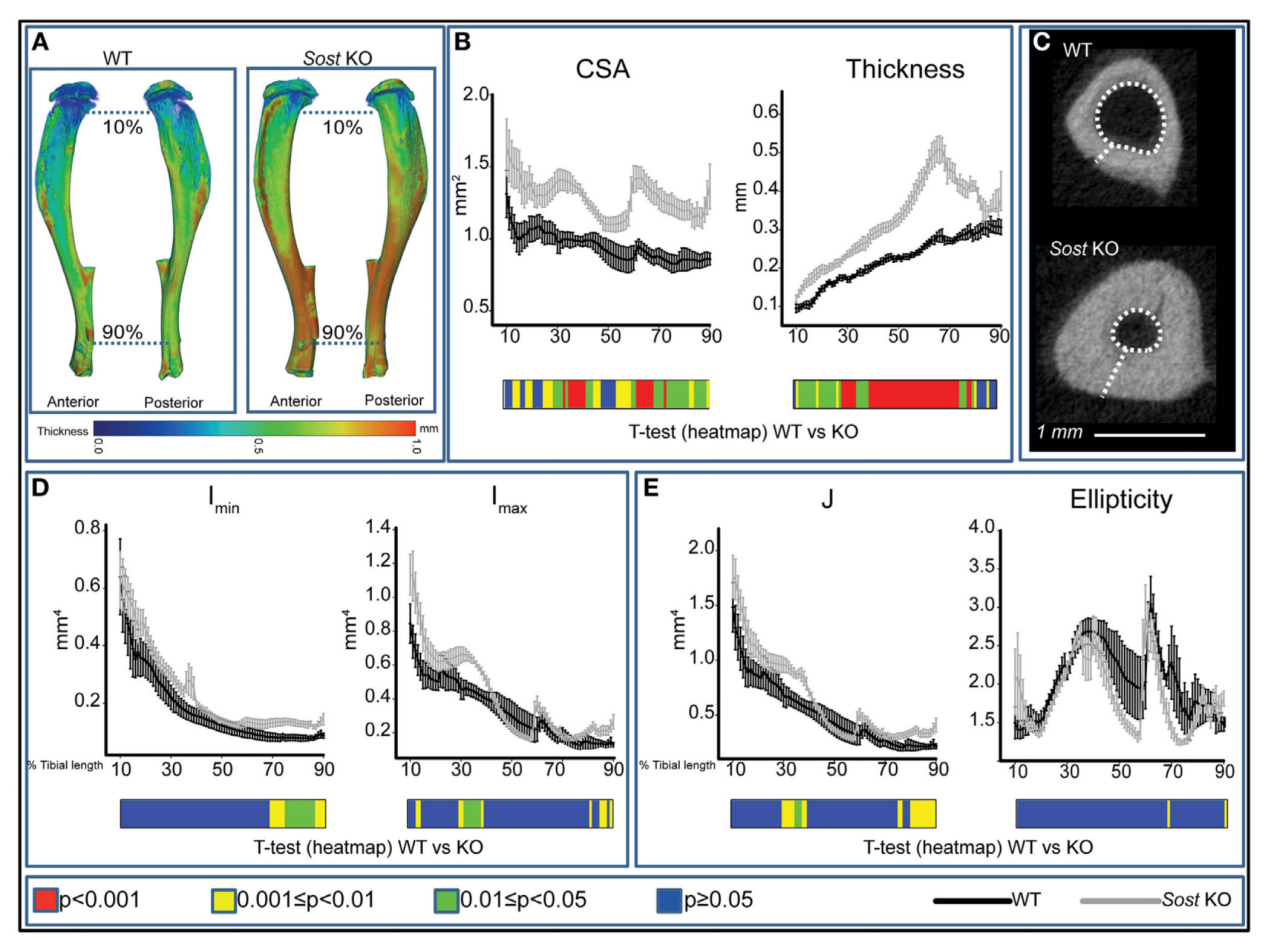
Modification in cortical bone phenotype in *Sost* knockout (KO) (gray) mice. **(A)** Representative 3D Micro-CT color-coded images of tibial cortical bone thickness. **(B)** Bone cross-sectional area (CSA) and mean cortical thickness between 10 and 90% of total tibial length. **(C)** Gross macroscopic comparison of microCT images of cortical tibia at the tibiofibular junction for both groups demonstrating a significantly thickened tibial cortex and reduced medullary cavity in *Sost* KO compared to wild-type (WT) mice. **(D)** Minimum and maximum second moments of area (I_min_ and I_max_ respectively) and **(E)** ellipticity and J (resistance to torsion) between 10 and 90% of total tibial length of WT and *Sost* KO mice. Graphical heat map **(B,D,E)** at the bottom of each graph summarizes statistical differences at specific matched locations along the tibial length, representative of overall effect of *Sost* deficiency: red *p* < 0.001, yellow 0.001 ≤ *p* < 0.01, green 0.01 ≤ *p* < 0.05, and blue *p* ≥ 0.05. Two-sample *t*-test was used to compare means between WT and *Sost* KO mice. Line graphs represent means ± SEM. Group sizes were *n* = 6 for WT and *Sost* KO mice.

We found that *Sost* deficiency did not alter tibial length (Table 1) but was a significant determinant of bone CSA (Figure 1B), producing higher bone CSA in *Sost* KO compared with WT mice along the entire tibia length. Furthermore, we found that *Sost* deficiency also contributed significantly to cortical thickness (Figures 1A,B), with *Sost* KO mice exhibiting higher thickness than WT mice. These increases in cortical thickness were accompanied by reduction in medullary cavity area (Figure 1C).

Our data showed that the overall effect of *Sost* deficiency on I_min_ was most pronounced distal to the mid-shaft, where significant increases in I_min_ were observed (Figure 1D), and that there was a lack of marked alteration in the proximal tibia. I_max_ was higher 30 and 85% along the tibia of *Sost* KO mice (Figure 1D). Predicted tibial resistance to torsion (J) is higher in *Sost* KO mice in two regions at ~25–35 and 70–90% of tibial length (Figure 1E). Moreover, we found that Sost deficiency did not modify tibial ellipticity, a measure of bone shape (Figure 1E).

### *Sost* Deficiency does not Alter Lacunar and Vascular Configurations

**Figure 2:**
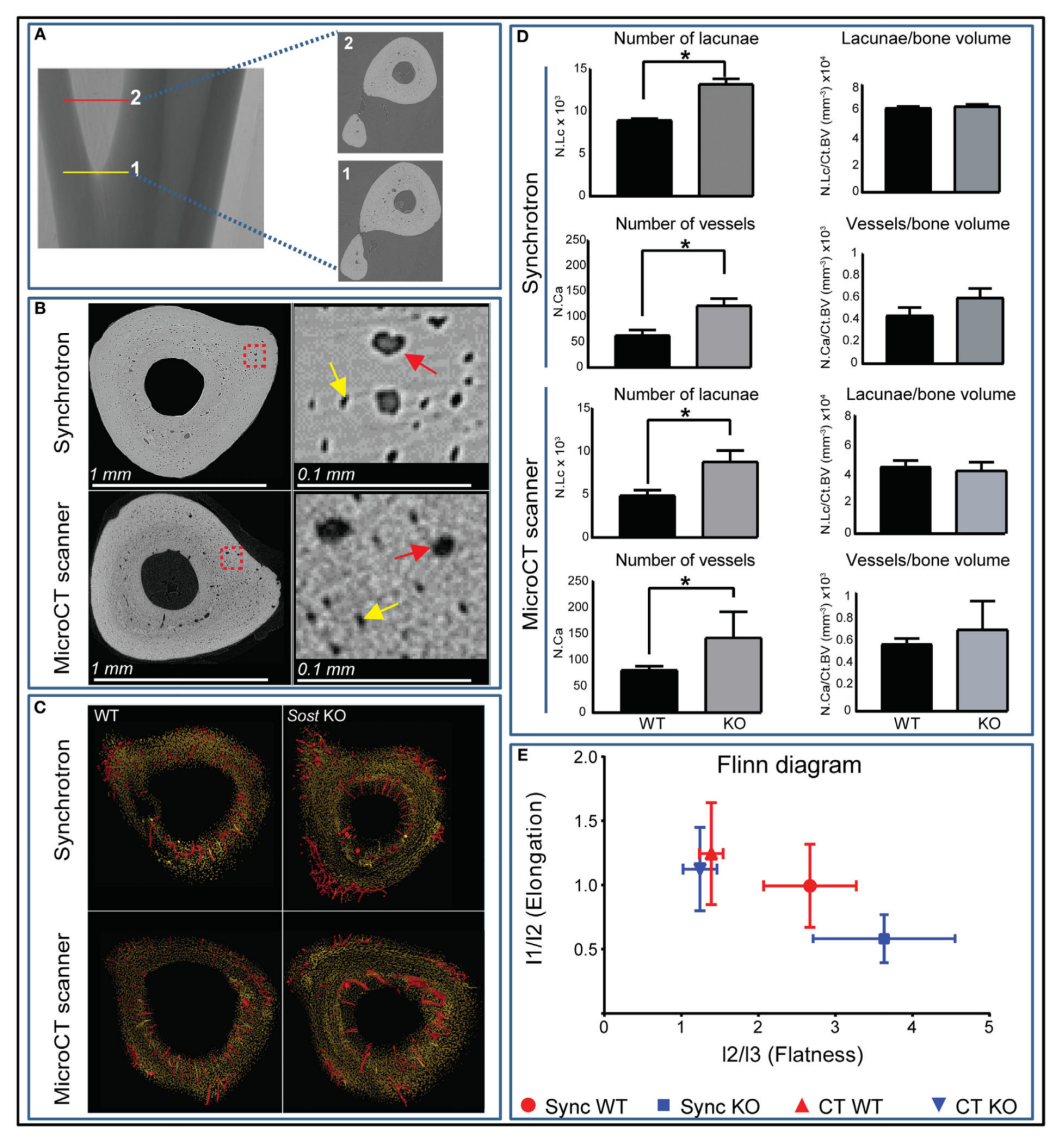
High-resolution nano-computed tomography (nanoCT) and synchrotron-based analysis of the cortex lacunar and vascular porosity at the tibiofibular junction. **(A)** Three 100 ascending images were selected from point 1 at the tibiofibular junction to point 2 and were used for porosity analysis. **(B)** Resolving power and gross macroscopic comparison of images obtained from the nanoCT and the synchrotron. Higher magnification quadrants are also shown right side of their respective images for better appreciation. Yellow and red arrows depict selected lacunar and canal pores in both imaging modalities. **(C)** Surface representation of the lacunar (yellow) and red (vascular canal porosity) segmented from 300 consecutive images from tibiofibular junction of wild-type (WT) and *Sost* knockout (KO) mice. **(D)** Number of lacunar pores and vascular canals of WT and *Sost* KO mice at 12 weeks of age. **(E)** Flinn diagram displaying lacunar shapes in WT and *Sost* KO tibia at tibiofibular junction. The *x*-axis represents lacunar flatness which was calculated by dividing lacunar intermediate radius (l2: length of best-fit ellipsoid’s intermediate radius) with lacunar minor radius (l3: length of best-fit ellipsoid’s minor radius). The *y*-axis represents lacunar elongation which was calculated by dividing lacunar major radius (l1: length of best-fit ellipsoid’s major radius) with lacunar intermediate radius (l2: length of best-fit ellipsoid’s intermediate radius). WT and *Sost* KO tibiae scanned at the synchrotron were from a separate batch compared to micro-CT and bench top nanoCT. Two-sample *t*-test was used to compare means between WT and *Sost* KO mice. Data represent means ± SEM with group sizes of *n* = 6 for WT and *Sost* KO mice.

Our data show that *Sost* deficiency leads to higher total volume (Tot.V) and bone cortical volume (Ct.BV) (*p* < 0.001; Table 1). Our morphometric evaluation of the cortical bone at the tibiofibular junction (Figures 2A,B), shows that absolute number of lacunar pores is greater in *Sost* KO than in WT bones, containing significantly greater total numbers of osteocyte lacunae (N.Lc; *p* < 0.001; Figures 2C,D) in images obtained from nanoCT and synchrotron. This greater lacunar number, however, was normalized to control WT mouse levels, when expressed per unit of bone volume (N.Lc/Ct.BV; Figure 2D) as well as total volume including bone, lacuna, and vascular canals volume (N.Lc/Tot.V; Table 1). The average lacunar volume (Lc.Avg.V; Table 1), total lacunar volume occupied by all lacunar pores (Lc.Tot.V; Table 1), and lacunar diameter (Lc.D; Table 1) were not significantly different in datasets of either imaging modalities between *Sost* KO and WT mice.

Greater absolute non-normalized N.Lc in *Sost* KO bone was also consistent with measures of greater N.Ca, in which significantly higher vascular canal number (N.Ca; *p* < 0.001; Figures 2C,D), were evident in *Sost* KO bones. This was also normalized to control WT mouse levels when total vascular canal number was expressed per unit bone volume (N.Ca/Ct.BV; Figure 2D) as well as total volume (N.Ca/Tot.V; Table 1). Furthermore, we found that *Sost* deficiency did not alter average volume of vascular canals (Ca.Avg.V; Table 1), total volume of all vascular canals (Ca.Tot.V; Table 1), or vascular canal diameter (Ca.D; Table 1). These data indicate that the *Sost* deficiency produces an elevation in bone volume but does not lead to modification in lacunar and vascular network density in bone.

Analysis of lacunar shape, using Flinn diagram (Flinn, 1962) (Figure 2E) showed that *Sost* deficiency does not modify osteocyte arrangement and shape. Lacunar elongation and flatness were not significantly altered in tibiofibular junction in *Sost* KO compared with WT in either imaging modalities. These data showed that *Sost* deficiency does not lead to divergence in osteocyte shape and organization in mouse tibiae.

## Discussion

Our data are consistent with previous studies (Krishnan et al., 2006; Li et al., 2008; Hassler et al., 2014; Suen et al., 2015; Kamiya et al., 2016; Qin et al., 2016; Shu et al., 2017) indicating that *Sost* deficiency leads to significant elevations of cortical bone mass. In addition, we find that these increases are not linked to significant changes in bone shape. Moreover, using two high-resolution imaging modalities, we examined whether the observed elevation of bone mass at macroscopic level in *Sost* KO mice extends to include differences in the lacunar and vascular composition of cortical bone. In particular, this focuses on the number and morphometric properties of the vascular and osteocyte lacunar cavities within the cortical bone in these mice.

We have previously used benchtop CT to study lacunar and vascular canal porosity using tibiofibular junction as a landmark (Javaheri et al., 2015). Benchtop nanoCT is essentially a micro-CT scanner with small enough focal spot, capable to be used at also sub-micrometer voxel size (0.6 μm/pixel). Herein, we have also employed synchrotron-based nanoCT imaging to compare and confirm suitability of using benchtop-based nanoCT imaging for porosity analysis. With both imaging modalities, the absolute numbers of lacunar and vascular cavities were significantly higher in *Sost* KO compared with WT mice at tibiofibular junction. This similarity suggests that data obtained from the benchtop CT are comparable, at least in terms of these parameters, to gold standard synchrotron imaging. We could not perform a paired comparison between benchtop and synchrotron nanoCT on the same specimens as bones scanned at the synchrotron were from a different batch.

Our detailed analyses reveal that there were no significant differences in shape, density or organization of lacunae or vascular canal porosity within the cortical bone of *Sost* KO mice. These data suggest that enhanced properties of bone on a macroscopic scale, specifically in terms of mass in *Sost*-deficient mice, are not reflected in significant alterations in the geometry and morphometry of lacunar and vascular porosity. The lacunar and vascular canal values per unit volume of bone obtained from both imaging modalities were similar to those reported previously (Carriero et al., 2014; Hemmatian et al., 2017). In addition, values relating to volume from both imaging modalities revealed that *Sost* deficiency does not alter average and total volume of lacunar or vascular canals. Diameter of lacunar and vascular canals were lower in KO compared with WT tibiae, but these differences did not reach levels of statistical significance. This may explain, minor and not major increases in total volume of lacunar and canal volumes despite having significantly higher absolute number of lacunar and canal cavities. The average volume of lacunar pores was in agreement with those reported previously (Wang et al., 2005; Schneider et al., 2007; Vatsa et al., 2008; Javaheri et al., 2015; Kerckhofs et al., 2016). The average lacunar volume (~360 μm^3^) we report in this manuscript is also in agreement with lacunar volume measurements reported by McCreadie et al. (2004) obtained using confocal microscopy.

Furthermore, when the absolute number of both lacunae and vessels are corrected for total and bone volume, the data become not significantly different between *Sost* KO and WT mice. This implies that the density of lacunae and vascular canals is unaffected by the action of SCLEROSTIN at least at this specific location in male mice at 12 weeks of age. These data suggest that the frequency of osteocyte recruitment from the osteogenic lineage is independent of SCLEROSTIN-mediated control, or alternatively that other factors ensure control of these recruitment processes and that there is, therefore, significant redundancy in this process. The statistically significant increases in bone mass in *Sost* KO mice implies, however, that this terminal stage of osteoblastic to osteocytic differentiation following entrapment in their own mineralized secretions (Dallas and Bonewald, 2010) occurs in a manner that is *Sost*-independent. Previous studies reported that the Wnt signaling and SCLEROSTIN regulate bone remodeling (Balemans et al., 2001; Van Wesenbeeck et al., 2003), influence pre-osteoblast differentiation and proliferation (Kato et al., 2002) and osteoblast-to-osteocyte transition *in vitro* (Atkins et al., 2011). We initially hypothesized that SCLEROSTIN influences the final stage of osteoblast differentiation in bone and expected the values related to osteocyte lacunae per unit bone volume in *Sost* KO mice to be significantly modified. As this does not appear to be the case, our data offer weight to the argument that any interaction between SCLEROSTIN and the maturation pathway occurs at an earlier stage in osteoblast cell lineage commitment. Similar arguments are also applicable to the role of SCLEROSTIN in vascular recruitment to bone. Further work should thus concentrate on identifying the exact stage or stages that are significantly influenced by the presence or absence of SCLEROSTIN.

There are several limitations to our study. The number of samples in our study are six per group and variation in our data, specifically the KO group (Table 1 and Figure 2D), might have influenced our findings. In this context, it is worth noting that murine bones lack Haversian canals and that it is possible that the role of SCLEROSTIN, in species where such canals comprise a significant volume of the cortex (humans), may differ (Perilli et al., 2015). In addition, we find that canal and lacunar porosity volumes recorded by benchtop CT are smaller than those revealed by synchrotron, by 40 and 35%, respectively. It is possible that despite the similar diameter of these pores with the two CT systems, there could be some artificially broken canals and hence smaller volume measured by the benchtop CT (Palacio-Mancheno et al., 2014).

In conclusion, our data show that *Sost* deficiency in mice, despite having a significant influence on the physical and structural properties of the whole bone does not modify lacunar and vascular canal porosities. Thus, SCLEROSTIN’s negative modulatory role on bone formation does not likely extend to a control over the terminal phase in osteocyte recruitment nor to any modification in bone’s vascularization in mice.

## Figures and Tables

**Table 1:** Porosity parameters representing lacuna and vascular porosity of male wild-type (WT) and *Sost* knockout (KO) mice at 12 weeks of age, detailing *t*-test comparisons for significant genotype effect of data obtained from nano-computed tomography (nanoCT) or synchrotron.

Morphometric parameters	WT synchrotron	KO synchrotron	*t*-Test	WT CT	KO CT	*t*-Test
**Bone parameters**						
Tibial length (mm)	17.39 ± 0.03	17.36 ± 0.03	NS	17.46 ± 0.04	17.23 ± 0.12	NS
Ct.BV (mm^3^)	0.146 ± 0.003	0.205 ± 0.006	<0.001	0.142 ± 0.002	0.209 ± 0.001	<0.001
Tot.V (bone + canal and lacunar pores) (mm^3^)	0.151 ± 0.003	0.211 ± 0.006	<0.001	0.145 ± 0.002	0.213 ± 0.001	<0.001
**Vascular canal**						
Ca.Avg.V (μm^3^)	4,529 ± 870	3,206 ± 1,201	NS	3,418 ± 671	2,444 ± 916	NS
Ca.Tot.V (mm^3^)	0.00035 ± 0.00004	0.00023 ± 0.00005	NS	0.00021 ± 0.00008	0.00028 ± 0.00002	NS
Ca.D (μm)	9.44 ± 0.81	7.93 ± 0.81	NS	8.21 ± 0.33	7.61 ± 0.24	NS
N.Ca/Tot.V (number/mm^3^)	443 ± 68	605 ± 605	NS	571 ± 45	691 ± 242	NS
**Lacunae**						
Lc.Avg.V (μm^3^)	396 ± 46	420 ± 31	NS	312 ± 19	288 ± 40	NS
Lc.Tot.V (mm^3^)	0.00581 ± 0.00002	0.00659 ± 0.00004	NS	0.00377 ± 0.00002	0.00428 ± 0.00001	NS
Lc.D (μm)	3.82 ± 0.28	3.39 ± 0.14	NS	3.13 ± 0.39	2.9 ± 0.21	NS
N.Lc/Tot.V (number/mm^3^)	63,189 ± 863	64,411 ± 1,240	NS	45,494 ± 4,170	42,761 ± 5,741	NS

Bone parameters included tibial length, cortical bone volume (Ct.BV), and total volume (Tot.V: volumes of bone plus lacunar and canal volumes). For vascular canals, average canal volume (Ca.Avg.V), total canal volume (Ca.Tot.V), canal diameter (Ca.D), and number of vascular canals per total volume (N.Ca/Tot.V) are shown. Morphometric measurements for lacunar pores, include average lacunar volume (Lc.Avg.V), total lacunar volume (Lc.Tot.V), lacunar diameter (Lc.D), and number of lacunar pores per total volume (N.Lc/Tot.V). WT and Sost KO tibiae scanned at the synchrotron were from a separate batch compared to micro-CT and benchtop nanoCT. Two-sample t-test was used to compare means between WT and Sost KO mice. Data represent means ± SEM with group sizes of n = 6 for WT and Sost KO mice.
